# The James Truman Dental Society of the University of Pennsylvania

**Published:** 1891-04

**Authors:** O. M. Brown


### THE JAMES TRUMAN DENTAL SOCIETY OF THE UNIVERSITY OF PENNSYLVANIA.

THE regular meeting of this society was held in the Library Building, Wednesday evening, January 21, 1891.

President J. A. McKee, Jr., in the chair.

Mr. Sydney F. Jacobi read a paper on

### PYORRHŒA ALVEOLARIS.

He maintained that the exciting cause of this disease, in at least ninety per cent. of cases, was due to a "deposit of serumic tartar" on the cervix and root of the tooth, this deposit being an abnormal exudation from the mucous glands in the free portion of the gum, which hugs the cervical portion of the tooth, and may be induced by any irritation of the tissues at this point.

Other causes he thought to be from mechanical or chemical irritation, such as irritation from a clamp, a bristle from a tooth-brush getting lodged in the gum, undue pressure of a plate against a tooth, or food allowed to ferment between the teeth, thus generating poisonous ptomaines which gain lodgement at the gingival border. Salivary calculus, he thought, had very little to do with causing the disease.

The treatment recommended was first to remove all deposits or other irritants, using great care not to injure the soft parts. Such tartar as could not be reached with scalers should be touched with acid to soften it and then washed away with a strong jet of water from a syringe.

The medical treatment to be that recommended by Dr. Truman, namely, first make an application of a twenty-five per cent. solution of sulphuric acid, using a thin orange-wood point to carry it up along the root as far as the disease has progressed. Apply to three teeth at a time and allow to remain a few seconds, then follow with bicarbonate of soda to neutralize the acid.

Next, wash out with tepid water, and pack the pockets with sulphate of quinia, which will excite healthy granulation.

He then described a bad case which he had treated successfully by this method, followed by a solution of chlorate of potassium as a mouth-wash after each meal and before retiring.

Tincture of myrrh was used in a weak solution two or three times a day.

Discussion was opened by Mr. Louis Stephan.

It was his opinion that tincture of myrrh should not be used in the treatment as it left a resinous precipitate in the mouth and around the teeth, which would hinder the healing.

Also would recommend the commercial sulphuric acid,—Mr. Jacobi having failed to state what preparation to use,—as the aromatic contained useless and harmful condiments.

He advised, in addition to the treatment prescribed, quinine internally (dose, four to six grains) in cases where a tonic was indicated.

He gave as additional causes which might produce the disease,—insoluable dentifrices, charcoal, tobacco, caustic agents, such as chloride of zinc, etc.

As all inflammatory conditions result in development of micro-organisms, he recommended that the use of clamps should be followed by an antiseptic agent, an excellent one being:

℞ Hydronaphthol, gr. v;

Alcoholis,

Aquæ dest., āā ℥i. M.

A good mode of treatment recommended by Dr. Harlan, consists in first filling the pockets with eucalyptus and iodoform. Allow this to remain a few days, after which the serumic deposits and all exfoliated bone may be removed, the pockets washed and then injected with hydrogen peroxide, which decomposes the pus and destroys micro-organisms.

After again drying the gums, inject the pockets with

Zinci iodinii, gr. xiv;
Aquæ, ℥i.

For chronic cases the proportion of iodide of zinc should be greater.

*Mr. Carpenter.*—I would like to ask if Mr. Jacobi or others here had any soreness following the application of the twenty-five-per-cent. solution of sulphuric acid?

*Mr. Jacobi.*—I think not. In a case I treated, of a gentleman who had cancer, he complained of increase of soreness after the application. As to the use of myrrh, it acted splendidly in my case. I saw the patient a few days ago, and found the gums much improved.

*W. H. Haines.*—I had a case last summer, for which I used the method described by Mr. Jacobi, and followed by a mouth-wash of tannic acid and listerine. The case improved, but did not get well, so I used iodine crystals, dissolved in creosote, applied around the teeth. This gave considerable pain, the teeth got loose, and the symptoms worse. I abandoned this and used eucalyptus oil and iodoform, followed by quinine, around the teeth. The case improved, but I had not produced a cure when I left home.

*Mr. Jacobi.*—Why did you use iodoform and creosote?

*Mr. Haines.*—It was recommended to me.

*The President.*—We have Dr. Darby with us; perhaps he will enlighten us.

*Dr. Darby.*—I was just wondering if those teeth would not have been loose, any way, if the gentleman had not used iodoform and creosote. I hardly think the creosote would cause loosening. In cases where the tissues are much inflamed, sulphuric acid will give severe pain for a time.

*Mr. Stephan.*—I think it would be a better plan to use the acid before removing the tartar, as the coating of tartar would save the teeth from injury by the acid.

*Dr. Darby.*—That would be good if the tartar covered the whole root, but it is only in nodules.

*Mr. Stephan.*—Are there not two kinds of tartar?

*Dr. Darby.*—The worst cases are where but one or two nodules are on the roots. There are some constitutions in which pyorrhœa is incurable, so you must not expect to cure all cases. If you save fifty per cent. you will be unusually successful. I have never seen a bad case where catarrh was not present.

Adjourned.

O. M. Brown.

